# Antioxidant capacities and total phenolic contents of 20 polyherbal remedies used as tonics by folk healers in Phatthalung and Songkhla provinces, Thailand

**DOI:** 10.1186/s12906-018-2131-y

**Published:** 2018-02-21

**Authors:** Wipawee Chanthasri, Nuntitporn Puangkeaw, Nongluk Kunworarath, Patcharawalai Jaisamut, Surasak Limsuwan, Katesarin Maneenoon, Piyapong Choochana, Sasitorn Chusri

**Affiliations:** 1 Natural Product Research Center of Excellence, Prince of Songkla University, Hat Yai, Songkhla, 90110 Thailand; 2 Faculty of Traditional Thai Medicine, Prince of Songkla University, Hat Yai, Songkhla, 90110 Thailand; 3 Faculty of Oriental Medicine, Rangsit University, Pathumthani, Thailand

**Keywords:** Antioxidant activity, Folkloric medicine, Traditional medicine, Tonics

## Abstract

**Background:**

Uses of polyherbal formulations have played a major role in traditional medicine. The present study is focused on the formulations used in traditional Thai folkloric medicine as tonics or bracers. Twenty documented polyherbal mixtures, used as nourishing tonics by the folk healers in Phatthalung and Songkhla provinces in southern Thailand, are targeted. Despite traditional health claims, there is no scientific evidence to support the utilization of polyherbal formulations.

**Methods:**

The phenolic and flavonoid contents of the polyherbal formulations and a series of antioxidant tests were applied to measure their capability as preventive or chain-breaking antioxidants. In addition, the cytotoxic activity of effective formulations was assayed in *Vero* cells.

**Results:**

Ninety-eight plant species belonging to 45 families were used to prepare the tested formulation. The preliminary results revealed that water extracts of THP-R016 and THP-R019 contain a high level of total phenolic and flavonoid contents and exhibit remarkable antioxidant activities, as tested by DPPH, ABTS, and FRAP assays. The extract of THP-R019 also showed the strongest metal chelating activities, whereas THP-R016 extract possessed notable superoxide anion and peroxyl radical scavenging abilities.

**Conclusions:**

The data provide evidence that the water extracts of folkloric polyherbal formulations, particularly THP-R016, are a potential source of natural antioxidants, which will be valuable in the pharmaceutical and nutraceutical industries. The free radical scavenging of THP-R016 may be due to the contribution of phenolic and flavonoid contents. Useful characteristics for the consumer, such as the phytochemical profiles of active ingredients, cellular based antioxidant properties and beneficial effects in vivo, are under further investigation.

**Electronic supplementary material:**

The online version of this article (10.1186/s12906-018-2131-y) contains supplementary material, which is available to authorized users.

## Background

Traditional medicine, especially herbal therapies, has a crucial role in the health care system in both developing and industrialized countries. According to recent survey results, the percentage taking herbal supplements was almost 20% in Europe [1]. In several countries, such as Nigeria [2], Turkey [3], Saudi Arabia [4], and Thailand [5], more than 40% of informants have used herbal medicine. It should be noted that 35% of respondents in the United States consumed herbal products as antioxidant supplements [6]. Free radicals have emerged as an important cause of oxidative stress-related diseases, including cardiovascular diseases, cancer, neurodegenerative disorders, and ageing. Effective antioxidants can delay or inhibit the oxidation of biomolecules, especially the polyunsaturated fatty acids of cell membranes, or prevent oxidative DNA damage, which affected several pathological conditions, including mutagenesis and carcinogenesis [7]. Therefore, herbal medicine may become a useful resource for new antioxidant compounds that can be used for the prevention and treatment of free radical-related diseases.

In recent years, several biological activities related to human health of traditional polyherbal formulations have been reported. The use of herbal-herbal combinations has been found in Ayurvedic [8], Chinese [9] as well as Thai traditional medicine practices [10]. However, the scientific evidence for potential bioactive compounds, therapeutic benefits, and safety information are limited. Recent studies have emphasized the antioxidant properties of polyherbal remedies prescribed in traditional medicine, such as *Sahatsatara* (Thai traditional medicine) [10], *Wuzi Yanzong* (Chinese medicine) [3], and *Charaka Samhita* (Ayurvedic medicine) [4]. Although several surveys on folk medicine in southern Thailand have previously been conducted [11–13], the evaluation of antioxidant activities of polyherbal formulation prescribed by folk healers has never been reported. Therefore, this study is focused on the assessment of antioxidant activities as well as total phenolic and flavonoid contents of 20 documented remedies that were selected based on their traditional uses as tonics or rejuvenators by folk healers in Phatthalung and Songkhla provinces [14, 15]. A series of antioxidant tests based on single electron transfer (SET) and hydrogen atom transfer (HAT) was used and the effective formulations were additionally tested for their toxicity effects against the *Vero* cell line.

## Methods

### Herbal materials

Twenty polyherbal remedies with either rejuvenating affects or used as health-promoting tonics were selected from prior studies, as summarized in Table 1. Herbal ingredients of the remedies were locally purchased from a licensed traditional medical drug store, Triburi Orsot in Songkla, Thailand. The plant materials were identified by a botanist, Assistant Professor Dr. Katesarin Maneenoon against reference specimens of the materia medica at the Faculty of Traditional Thai Medicine, Prince of Songkla University, Thailand. The plant parts were washed with sterile distilled water, oven-dried at 60 °C for 72 h, and then pulverized. The powdered herb was passed through a 16-mesh sieve, weighed, and stored in vacuum-sealed bags at 4 °C until further use. The mixtures were formulated using different proportions of different types of plant parts, as described in Additional file 1: Table S1.

**Table 1:** Polyherbal formulas and their medical uses

Remedies	Method of preparation and administration	Traditional uses
THP-R001^a^	Pounded, dried, mixed with honey and made into boluses	refreshing and rejuvenating effect on the entire body; reduces joint and muscle pain and flatulence
	Take one bolus twice a day before meals	
THP-R002^a^	Pound, mixed with boiled water or honey and made into boluses; take 4–5 boluses thrice a day before meals	nourishes the skin; promotes blood circulation and used as a blood tonic
THP-R003^a^	Pounded, dried, mixed with honey and made into boluses; take 2–4 boluses twice a day before meals	health tonic and promotes longevity
THP-R004^a^	Pounded, dried, mixed with honey and made into boluses; take one bolus once a day before meals	health tonic, skin refreshment, and promotes longevity
THP-R005^b^	Dried and boiled with water; drink 40–50 mL thrice a day before meals	carminative and a tonic
THP-R006^b^	Pounded, dried, mixed with honey and made into boluses; take two boluses twice a day after meals	stimulating and rejuvenating the entire body
THP-R007^b^	Pounded and infused in hot water (90–97 °C, 3–5 min); drink 100–150 mL of the infusion twice a day after meals	health promoting and hypolipidemic agents
THP-R008^c^	Dried, boiled with water, and filtered the infusion; drink 50–60 mL twice a day after meals	used as a health tonic and used to treat fatigue
THP-R009^c^	Dried, boiled with water, and filtered the infusion or pounded, dried, mixed with honey and made into boluses; drink 50–60 mL or take two boluses twice a day after meals	health tonic, sex stimulant, or sexual performance enhancer
THP-R010^d^	Extracted by boiling water for at least 3 h; drink 40–50 mL thrice a day before meals	an appetite inducer and tonic
THP-R011^e^	Pounded, dried, and made tinctures (alcohol extract) or mixed with honey and made into boluses; drink 30 mL or take three boluses once a day before meals	nourishing and tonifying agents; reduces joint and muscle pain, and applied as a sex stimulant
THP-R012^f^	Dried, boiled with water, and filtered the infusion; drink 40–50 mL thrice a day before meals	improvement blood circulation tonic
THP-R013^g^	Extracted by boiling water for at least 3 h; drink 10–20 mL thrice a day before meals	stimulates and rejuvenates the entire body
THP-R014^h^	Dried, boiled with water, and filtered the infusion; drink 5 mL thrice a day before meals	health tonic and sex stimulant; reduces joint and muscle pain and lowers blood glucose
THP-R015^h^	Extracted by boiling water for 15 min; drink 5 mL thrice a day before meals	tonic, sexual performance enhancer, and lowering blood glucose
THP-R016^h^	Pounded, dried, and mixed approximately 5 g with 100 mL of warm water; drink once a day before meals	stimulate and rejuvenate the entire body
THP-R017^i^	Pounded, dried, and mixed approximately 5 g with 100 mL of warm water; drink once a day before meals	blood tonic, reduce joint and muscle pain
THP-R018^j^	Extracted by boiling water for at least 3 h and filtered the infusion; drink 50 mL thrice a day before meals	stimulate and rejuvenate the entire body
THP-R019^k^	Pounded, dried, extracted by boiling in water, and filtered the infusion; drink 50 mL once a day before meals	blood tonic; reduce joint and muscle pain
THP-R020^k^	Pounded, dried, and made tinctures (water extract); drink 50 mL once a day before meals	health tonic

The traditional healers who prescribed the remedies were ^a^Mr. Prayut Boonyug, ^b^Mr. But Parnpradit, ^c^Mr. Yayo Lumkun, ^d^Mr. Num Nokkeaw, ^e^Mr. Prasert Kaewpradit, ^f^Mr. Nhom Ratchgaew, ^g^Mr. Somporn Chanwanisakul, ^h^Mr. Yop Lomsa, ^i^Mr. Lep Boonmee, ^j^Mr. Kart Eatmong, and ^k^Mr. Rowan Watjirasoporn

### Preparation of polyherbal extracts

To prepare the extract, 100 g of powdered herbal mixture were boiled in 1000 mL of water at 96.3 ± 0.6 °C for 20 min. The resultant extracts were filtered through a Whatman No. 1 filter paper. The dried extracts were obtained using the freeze-drying process (freeze dryer) and their yields were calculated with respect to the starting materials (Table 3). Each extract was then dissolved in ethanol (25 mg/ml) for phytochemical and biological analyses. The entire study was performed using one batch of the crude extracts to manipulate batch-to-batch variation and maximize the consistency of the polyherbal extracts.

### Quantification of phytochemical contents

#### Total phenolic content (TPC)

The TPC of each polyherbal extract was quantified based on the Folin-Ciocalteu method according to Abbasi et al. [16] with few modifications. Briefly, 120 μL of the extract (2.5 mg/mL) was mixed with 1 mL of 10-fold diluted Folin-Ciocalteu reagent for 5 min, which was followed by the addition of sodium carbonate solution (Ajax Finechem, New Zealand) (1 mL; 20%*w*/*v*). The mixture was then thoroughly mixed and kept in the dark at room temperature for 90 min before the absorbance was read at 725 nm (Sunrise™ Microplate reader, Tecan Group Ltd., Switzerland). The TPC was expressed as milligrams of gallic acid (Sigma-Aldrich Chemie, Germany) equivalent per gram of extract.

#### Total flavonoid content (TFC)

The TFC in the studied samples was estimated by the aluminium chloride colorimetric method as described by Abbasi et al. (2015) [16], with minor modifications. In brief, 50 μL of the extract (2.5 mg/mL) was mixed with 4 mL of distilled water, 300 μL of 5% (*w*/*v*) of sodium nitrite (Ajax Finechem, New Zealand), and 300 μL of 10% (w/v) aluminium trichloride (Ajax Finechem, New Zealand). The mixture could stand for 6 min at room temperature; then, 2 mL of sodium hydroxide (1 M) was added to stop the reaction. The final volume of the mixture was adjusted to 10 mL with sterile-distilled water and the absorbance was measured at 510 nm after 10 min against the reagent blank. The TFC was calculated from a calibration curve using catechin standard solution, and the result was mentioned as milligrams of catechin equivalent per gram of extract.

### Gas chromatography-mass spectrometry analysis of essential oils from some herbal ingredients of an effective formula, THP-R016

Three active herbal ingredients of an effective formula, THP-R016 including *Alpinia galanga, Cyperus rotundus,* and *Piper retrofractum* were submitted to hydrodistillation at 100 °C for 5 h, using a Clevenger-type apparatus. GC-MS analysis of the essential oils were done on a TRACE™ GC Ultra system (Thermo Scientific, Waltham, MA) that was attached to an ISQ™ Series spectrometer. The gas chromatograph was equipped with a TRACE™ TR-1MS fused-silica capillary column (30 m × 0.25 mm id, film thickness 0.25 μm; Thermo Scientific, San Jose, CA). The GC oven was held at 60 °C for 3 min then ramped to 300 °C at 5 °C/min; inlet temperature, 280 °C; carrier gas, 1.0 mL/min Helium; injection volume, 1 μL; split ratio, 10:1. The GC to MS transfer line was maintained at 300 °C and the mass spectrometer was carried out in electron ionisation mode with an ion source temperature of 250 °C; solvent delay, 3 min. All data were acquired by collecting the full-scan mass spectra within a scanned mass range of 10–400 amu.

### Metal-chelating activity (MCA)

The chelation of ferrous ions by the polyherbal extracts was measured using the methods described by Wong et al., (2014) [17]. Aliquots (250 μL) of two-fold dilutions of the extract (0.03–62.50 mg/mL) were mixed with 800 μL of distilled water and 25 μL of iron (II) chloride (2 mM). The reaction was then initiated by the addition of 50 μL of ferrozine (5 mM). After the incubation at room temperature for 10 min, the absorbance of the stable ferrous-ferrozine complex was monitored at 562 nm. The metal chelator, EDTA, was used as a positive control. The percentage chelating capacity was expressed as follows:

$$ \frac{\mathrm{MCA}\ \left(\%\right)=\kern1em \left({\mathrm{OD}}_{\mathrm{control}}-{\mathrm{OD}}_{\mathrm{sample}}\right)\times 100}{{\mathrm{OD}}_{\mathrm{control}}} $$

The MCA values of the extracts and EDTA were expressed as the 50% inhibition concentration of the ferrous ion ferrozine complex (IC_50_; mg/mL).

### Antioxidant properties

#### DPPH and ABTS radical scavenging assays

The radical scavenging ability with DPPH radical was determined according to a previously published method [17]. In brief, a volume of 20 μL of each sample at different concentrations (2-fold dilution; 2500–1.22 μg/mL) was mixed with 180 μL of 80 μM DPPH solution in ethanol in a 96-well plate. The plate was shaken and allowed to reach a steady state at room temperature in the dark for 30 min. DPPH bleaching was measured by monitoring the absorbance at 520 nm.

The scavenging activity of the extracts on ABTS radical cation (ABTS^+^) was based on the method by Wong et al. [17] with slight modification. Briefly, ABTS^+^ solution was prepared by adding 2 mM ABTS to 2.45 mM potassium persulfate in a volume ratio of 1:1. The mixture was incubated in the dark at room temperature for 16 h, and it was then diluted with ethanol to an absorbance of 0.70 ± 0.05 at 734 nm. After the addition of 10 μL of each extract at different concentrations (2-fold dilution; 2500–1.22 μg/mL) to 1 mL of the diluted ABTS^+^ solution, the absorbance at 734 nm was measured at six minutes after initial mixing.

Appropriate blank measurements were performed and Trolox was used at a positive control. DPPH/ABTS^+^ based-scavenging activities (%) were calculated using the following equation:

$$ \frac{\mathrm{Scavenging}\ \mathrm{activity}\ \left(\%\right)=\kern1.5em \left({\mathrm{OD}}_{\mathrm{control}}-{\mathrm{OD}}_{\mathrm{sample}}\right)\times 100}{{\mathrm{OD}}_{\mathrm{control}}} $$

The scavenging ability of the extracts and Trolox were expressed as inhibition concentration (IC_50_; mg/mL), causing 50% inhibition of DPPH/ABTS^+^ radicals.

#### Ferric-reducing antioxidant power (FRAP) assay

The ferric ion reducing power of the extracts was performed according to the procedure described in the literature (Abbasi et al., 2015) [16], which was based on the reduction of ferric-tripyridyl triazine (TPTZ) complex to ferrous-TPTZ that was formed in the presence of electron donating antioxidants at low pH. The FRAP reagent was freshly prepared by mixing 10 mL of 300 mM acetate buffer, 1 mL of 10 mM TPTZ solution, and 10 mL of 20 mM ferric chloride. The tested extracts were diluted in ethanol at a concentration of 0.625 mg/mL. An aliquot of 150 μL of the extracts was mixed with 1.35 mL of the FRAP reagent and then placed at 37 °C for 30 min in the dark. The absorption of an intense blue colour complex of ferrous-TPTZ in the reaction mixture was monitored at 596 nm. The calibration curve was obtained by plotting the absorbance at 596 nm versus ethanol solutions of known ferrous concentrations. The reducing capacity of each extract was expressed as micromoles of ferrous per milligram of extract (μM Fe_2_SO_4_/mg extract).

#### Superoxide anion radical scavenging assay

The percentage inhibition of superoxide anion generation by eight effective formulations was based on the reduction of nitroblue tetrazolium (NBT). Superoxide radical is generated by the riboflavin/methionine/ illuminate system and assayed by the reduction of NBT to form a purple-coloured formazan (NBT^2+^) caused by the generated superoxide radicals. Briefly, 100 μL of NBT (400 μg/mL) was added to 0.4 mL of the reaction mixture containing riboflavin (30 μg/mL), methionine (30 μg/mL), EDTA (20 μg/mL), and extract (freshly prepared in 0.05 M phosphate buffer, pH 7.4) at various concentrations (2-fold dilution; 156.25–4.88 μg/mL). The photoinduced reactions were performed by illumination at 25 °C for 25 min using fluorescent lamps (20 W). The absorbance of formazan dye was measured at 560 nm against an appropriate blank solution. Catechin was used as a positive control. The capability to scavenge superoxide radical was calculated using the equation mentioned above and expressed as the inhibition concentration (IC_50_; mg/mL) causing 50% inhibition of superoxide anion radicals.

#### Peroxyl radical scavenging assay

The assay was modified from the method described by Gillespie et al. [18] in black round bottom 96-well microplates using Trolox as a control standard. Different concentrations of the effective extracts and positive control (2-fold dilution; 100–0.2 μg/mL), fluorescein (0.4 nM), and 2,2′-Azobis(2-amidinopropane) dihydrochloride (AAPH) (153 mM) were prepared in 75 mM phosphate buffer (pH 7.4). Twenty-five μL of each tested solution was mixed with 150 μL of the fluorescein solution and incubated for 30 min at 37 °C. An aliquot of 25 μL of AAPH solution was added, and the decay of fluorescence at 528 nm was immediately monitored with excitation at 485 nm every 5 min for 90 min. The antioxidant capacities of the extracts were expressed as μM of Trolox equivalent per μg of the extract (μM of TE/μg of E).

### Cytotoxic effects

The extracts of effective formulations were further tested for in vitro cytotoxicity on the Vero cell line by green fluorescent protein (GFP)-based assay, which was done by the National Center for Genetic Engineering and Biotechnology, National Science and Technology Development Agency, Pathumthani, Thailand (http://www.biotec.or.th/bioassay/). Ellipticine was used as a positive control.

### Statistical analysis

Antioxidant activities were analysed in triplicate in at least three different experiments and their results were presented as the mean ± SD. Statistical analyses of the data were performed using the Statistical Package for the Social Sciences software (SPSS 17) for Windows. The correlation between parameters was performed by Pearson’s correlation test and *p* values less than 0.05 were considered significant. The results were analysed by Student’s t-test for comparison between two means. One-way ANOVA with Tukey’s HSD as post hoc tests were used to assess differences in multiple samples. A difference was considered statistically significant for *p* values less than 0.05.

## Results

### Antioxidant related phytochemicals

**Fig. 1:**
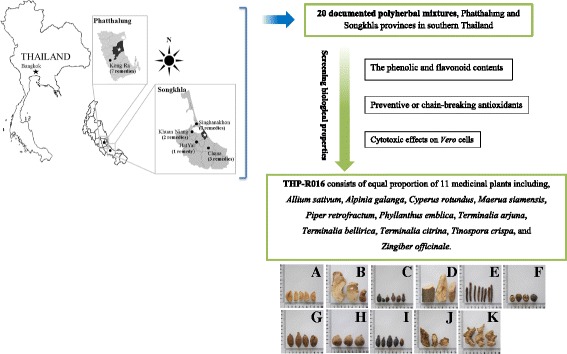
Map showing the area that herbal remedies used in this study have been found (a blank map of Thailand and regions map of Thailand were taken from http://commons.wikimedia.org with modification),THP-R016 consists of equal proportion of 11 medicinal plants including, *Allium sativum* L. (**a**), *Alpinia galanga* (L.) Willd. (**b**), *Cyperus rotundus* L. (**c**), *Maerua siamensis* (Kurz) Pax (**d**), *Piper retrofractum* Vahl (**e**), *Phyllanthus emblica* L. (**f**), *Terminalia arjuna* (Roxb. Ex DC.) Wight & Arn. (**g**), *Terminalia bellirica* (Gaertn.) Roxb. (**h**), *Terminalia citrina* (Gaertn.) Roxb. ex Fleming (**i**), *Tinospora crispa* (L.), Hook. f. & Thomson (**j**), and *Zingiber officinale* Roscoe (**k**)

Selection of polyherbal formulas to evaluate the antioxidant properties and phenolic contents is based on their traditional use as tonics, rejuvenators, nourishments, or bracers. Thirteen formulas are prescribed by seven folk healers from Songkhla province, and seven formulas are used by four folk healers from Phatthalung province (Fig. 1). As shown in Table 1, decoction and paste were the major modes of preparation; therefore, the hot water extraction method was selected to mimic these traditional medicinal uses of the tested remedies. Extraction yields obtained for the formulation extracts ranged from 2.67–11.83%. The results of this experiment revealed that maximum levels of TPC were found in the water extract of THP-R016 (384.6 ± 3.1 mg GAE/g extract), which was followed by THP-R019 (347.4 ± 12.0 mg GAE/g extract), THP-R010 (269.8 ± 11.1 mg GAE/g extract), THP-R015 (211.0 ± 2.9 mg GAE/g extract), and THP-R014 (207.2 ± 8.2 mg GAE/g extract), while the minimum value was observed in THP-R001 (39.3 ± 5.3 mg GAE/g extract). The TFC of the studied samples are presented in Table 2, indicating that THP-R019 extract exhibits the highest value (225.3 ± 2.4 mg CAE/g extract), which was followed by THP-R017 (132.7 ± 1.5 mg CAE/g extract), THP-R010 (131.2 ± 1.5 mg CAE/g extract), and THP-R014 (103.0 ± 3.1 mg CAE/g extract), whereas minimum values were noted for THP-R020 (34.2 ± 1.5 mg CAE/g extract).

**Table 2:** Total phenolic and total flavonoid contents of traditional polyherbal formulas

Samples	Phenolic content	Flavonoid content
	mg gallic acid/g extract TPC	mg catechin/g extract TFC
THP-R001	39.3 ± 5.3	37.7 ± 1.8
THP-R002	134.1 ± 6.3	63.4 ± 2.3
THP-R003	114.1 ± 7.1	51.6 ± 0.9
THP-R004	151.9 ± 4.6	68.4 ± 1.0
THP-R005	107.7 ± 4.8	60.8 ± 0.2
THP-R006	77.3 ± 4.6	41.9 ± 0.1
THP-R007	93.8 ± 2.7	43.4 ± 0.1
THP-R008	109.7 ± 6.8	55.3 ± 2.3
THP-R009	72.5 ± 3.1	40.1 ± 0.3
THP-R010	269.8 ± 11.1	131.2 ± 1.5
THP-R011	106.9 ± 5.1	64.7 ± 2.0
THP-R012	150.7 ± 7.5	80.9 ± 0.8
THP-R013	81.7 ± 6.1	61.3 ± 3.1
THP-R014	207.2 ± 8.2	103.0 ± 3.1
THP-R015	211.0 ± 2.9	42.9 ± 0.9
THP-R016	384.6 ± 3.1	51.9 ± 0.7
THP-R017	148.0 ± 9.5	132.7 ± 1.5
THP-R018	62.1 ± 3.4	37.0 ± 0.2
THP-R019	347.4 ± 12.0	225.3 ± 2.4
THP-R020	57.0 ± 3.4	34.2 ± 1.5

### Ability of extracts to act as preventive and chain-breaking antioxidants

THP-R009 exhibited the highest MCA (IC_50_; 0.3 mg/mL) where THP-R011, THP-R013, THP-R014, THP-R017, and THP-R020 were also found to possess the chelating activities (IC_50_; 0.48–0.58 mg/mL), but these activities were lower than that of EDTA. In this study, a negative correlation between phytochemical contents (TPC and TFC) and ferrous ion chelating abilities were observed (Table 4). The antioxidant capacities of the herbal formula extracts was further measured by the reduction of exogenous free radicals using DPPH, ABTS, and FRAP methods. The results of ferric reducing capacities of the extracts are given in Table 3. Among all tested extracts, THP-R019 had the highest FRAP value (96.86 ± 1.63 μM Fe_2_SO_4_/mg extract), which was followed by THP-R016 (95.76 ± 2.35 μM Fe_2_SO_4_/mg extract), THP-R012 (87.12 ± 1.15 μM Fe_2_SO_4_/mg extract), and THP-R015 (84.22 ± 1.23 μM Fe_2_SO_4_/mg extract), respectively. The radical scavenging effects of the sample extracts were primarily assessed using the DPPH and ABTS assays. The trend for these radical scavenging activities of the 20 tested extracts did not vary markedly from their ferric reducing capacities. Similar to the results obtained from FRAP assays, THP-R016 and THP-R019 extracts showed very strong scavenging activities against both DPPH and ABTS radicals. The IC_50_ value of THP-R016 extract in the ABTS assay was 0.34 mg/mL, which was comparable to the value of Trolox. The IC_50_ value of this extract on DPPH radicals was 2 times more than that of the standard.

**Table 3:** Extraction yields and antioxidant capacities of polyherbal formulas

Samples	Yields	MCA^a^	FRAP^b^ assay	Radical scavenging activities; IC_50_ (mg/mL)	
	% (*w*/w)	IC_50_ (mg/mL)	μM Fe_2_SO_4_/mg extract	DPPH	ABTS
THP-R001	5.68	0.93 ± 0.00	11.61 ± 0.25	–	–
THP-R002	4.60	0.94 ± 0.01	39.79 ± 0.72	2.15 ± 0.41	1.15 ± 0.00
THP-R003	5.68	0.63 ± 0.02	18.58 ± 0.35	–	2.01 ± 0.03
THP-R004	3.99	0.80 ± 0.01	42.51 ± 0.76	0.92 ± 0.02	1.09 ± 0.06
THP-R005	10.54	0.84 ± 0.01	38.84 ± 0.78	1.61 ± 0.03	1.38 ± 0.04
THP-R006	8.93	0.64 ± 0.01	15.63 ± 0.30	–	1.96 ± 0.10
THP-R007	8.07	0.64 ± 0.01	20.86 ± 0.65	–	1.45 ± 0.05
THP-R008	10.95	0.88 ± 0.01	51.62 ± 0.46	1.77 ± 0.05	1.31 ± 0.03
THP-R009	11.83	0.30 ± 0.01	20.27 ± 0.46	2.42 ± 0.05	1.79 ± 0.02
THP-R010	8.26	2.81 ± 0.04	87.12 ± 1.15	0.53 ± 0.02	0.42 ± 0.00
THP-R011	8.52	0.58 ± 0.02	30.33 ± 0.65	2.10 ± 0.03	1.31 ± 0.01
THP-R012	9.04	1.46 ± 0.06	40.70 ± 0.95	1.04 ± 0.07	0.87 ± 0.01
THP-R013	10.07	0.49 ± 0.01	20.92 ± 0.27	2.22 ± 0.04	1.89 ± 0.05
THP-R014	2.67	0.58 ± 0.02	54.55 ± 1.24	1.43 ± 0.07	0.55 ± 0.01
THP-R015	5.52	1.21 ± 0.01	84.22 ± 1.23	0.43 ± 0.02	0.70 ± 0.01
THP-R016	10.96	1.27 ± 0.02	95.76 ± 2.35	0.16 ± 0.00	0.34 ± 0.00
THP-R017	4.60	0.58 ± 0.02	82.32 ± 0.81	1.51 ± 0.24	1.07 ± 0.01
THP-R018	3.13	0.95 ± 0.02	18.75 ± 0.42	–	2.46 ± 0.10
THP-R019	4.31	1.24 ± 0.03	96.86 ± 1.63	0.50 ± 0.02	0.40 ± 0.01
THP-R020	4.09	0.57 ± 0.01	18.57 ± 0.15	–	–
Trolox	–	–	–	0.09 ± 0.01	0.34 ± 0.01
EDTA (μg/mL)	–	8.48 ± 0.34	–	–	–

-; Not applicable

^a^ MCA; metal chelating activity

^b^FRAP; ferric ion reducing antioxidant power

### Correlation between free radical inhibition activity and phenolic compounds

The Pearson’s correlation coefficients were evaluated to describe the inter-relationships of the results obtained with the different methods in the polyherbal formulation extracts (Table 4). There were significantly high positive correlations between antioxidant properties and TPC. In addition, the results revealed that there were moderate positive correlations between TFC and the antioxidant properties.

**Table 4:** The correlation analysis of total antioxidant capacities and main active ingredients of water extracts prepared from 20 polyherbal medicines used as rejuvenators

	Pearson’s correlation (*p* value)				
	ABTS^a^	FRAP^a^	MCA^a^	TPC^b^	TFC^b^
DPPH^a^	0.959 (0.000)	0.884 (0.000)	−0.690 (0.001)	0.882 (0.000)	0.592 (0.006)
ABTS		0.897(0.000)	−0.711 (0.000)	0.939 (0.000)	0.695 (0.001)
FRAP			−0.734 (0.000)	0.895 (0.000)	0.699 (0.001)
MCA				−0.751 (0.000)	−0.449(0.047)
TPC					0.682 (0.001)

^a^Antioxidant capacities of the extracts were expressed in terms of their inhibitory activity against ABTS• + and DPPH radicals (% inhibition at 1.2 mg/mL), ferric reducing antioxidant power (FRAP; μM Fe_2_SO_4_/mg extract), and ferrous ions metal chelating activity (MCA; % chelating activity at 3.5 mg/mL)

^b^Total phenolic content (TPC) and total flavonoid content (TFC) expressed as mg of gallic acid equivalent per g extract and mg catechin equivalent per g extract, respectively

### Inhibitory activity of extracts on superoxide anion radicals and cytotoxic effects

**Fig. 2:**
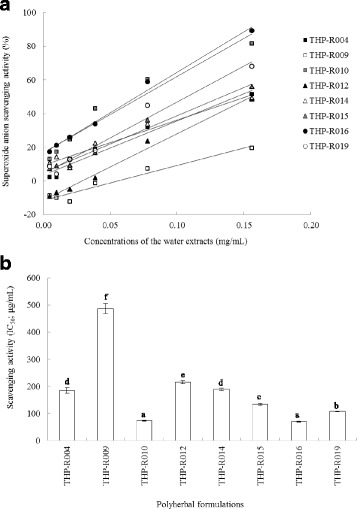
Superoxide anion scavenging activity of water extracts prepared from Thai traditional polyherbal formulations. The water extracts inhibited superoxide anion production in a dose-dependent manner (**a**). The IC_50_ values (50% inhibitory concentration) are expressed as the mean ± SD (**b**). The IC_50_ value of catechin, a positive control, was 9.1 ± 0.3 μg/mL. Different letters indicate statistically significant differences (*p* < 0.01)

As presented in Fig. 2a, the tested extracts scavenged superoxide anion in the riboflavin/methionine/illuminate system in a concentration-dependent manner. The IC_50_ values of THP-R004, THP-R009, THP-R010, THP-R012, THP-R014, THP-R015, THP-R016, and THP-R019 for scavenging the free radicals were found to be 184.7 ± 10.4, 486.6 ± 18.9, 73.6 ± 1.0, 215.3 ± 5.6, 189.0 ± 4.9, 133.8 ± 2.7, 70.0 ± 1.2, and 107.7 ± 1.0 μg /mL, respectively. The IC_50_ values of THP-R010 and THP-R016 were significantly less than that of the other extracts, which clearly indicated their greater efficiency as superoxide anion scavengers (Fig. 2b). Moreover, the non-cytotoxic effect was observed in normal cell lines when the cells were incubated for 24 h with THP-R004, THP-R015, and THP-R016. These effective formulas have an IC_50_ greater than 50 μg/mL against *Vero* cells, these values were found to be higher than a positive control, ellipticine (IC_50_ value of 0.68 μg/mL).

### Peroxyl radical scavenging activity of an effective remedy, THP-R016

**Fig. 3:**
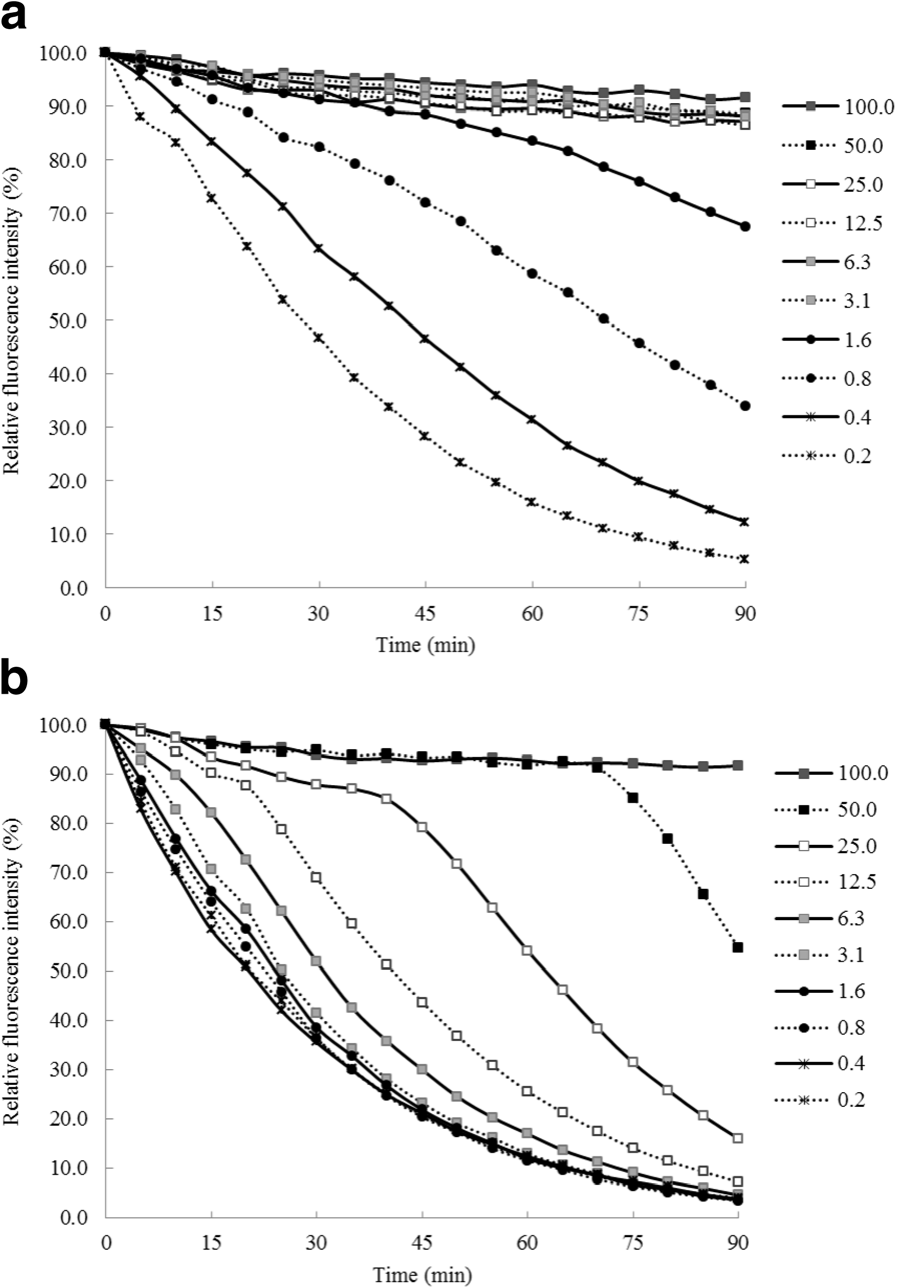
Anti-peroxyl radical activity, fluorescence decay curves of fluorescein in the presence of THP-R016 water extract (**a**) and the reference antioxidant trolox (**b**)

The present study shows that the fluorescence signal of fluorescein in the assay solutions of THP-R016 at concentration ranging from 3.1 to 100.0 μg/mL and trolox at 100 μM was stable for 90 min (Fig. 3). In this study, THP-R016 exhibits peroxyl radical scavenging properties with an ORAC value of 705.48 ± 33.44 μM Trolox/μg of extract. The GC-MS analysis (Table 5) revealed the major constituents as caryophyllen, pentadecane, 1,4,7,-cycloundecatriene, 1,5,9,9-tetramethyl-, Z,Z,Z-, 8-heptadecene, and heptadecane for the essential oil extracted from *Piper retrofractum*; 4-isopropyl-1-methoxy-1,6-dimethyl-1,2,3,4-tetrahydronaphthalene, copaene, α-cubebene, β-calacorene, and 11-hydroxy-2,3,4,11-tetrahydro-6H-pyrimido[2,1-b]quinazolin-6-one for *Cyperus rotundus*; and β-bisabolene, cis-α-bergamotene, β-sesquiphellandrene, 1,8-cineole, and chavicol, acetate for *Alpinia galangal*.

**Table 5:** Phyto-constituents identified in three essential oils of *Piper retrofractum* Vahl., *Alpinia galanga* L., and *Cyperus rotundus* L

Medicinal plants	Yield	RT	Compound names	Match Factor	Formula	MW	%Peak area
*Alpinia galanga* L.	0.43	20.72	β-Bisabolene	95.7	C_15_H_24_	204	14.0
		18.91	cis-α-Bergamotene	96.6	C_15_H_24_	204	10.0
		21.06	β-Sesquiphellandrene	94.0	C_15_H_24_	204	9.7
		8.08	1,8-Cineole	97.3	C_10_H_18_O	154	6.0
		16.55	Chavicol, acetate	98.8	C_11_H_12_O_2_	176	4.3
*Cyperus rotundus* L.	1.02	21.31	4-Isopropyl-1-methoxy-1,6-dimethyl- −1,2,3,4-tetrahydronaphthalene	90.3	C_15_H_22_	202	11.7
		17.52	Copaene	95.2	C_15_H_24_	204	5.0
		16.81	α-Cubebene	94.5	C_15_H_24_	204	4.7
		21.69	β-Calacorene	89.9	C_15_H_20_	200	4.5
		21.61	11-hydroxy-2,3,4,11-tetrahydro-6H-pyrimido[2,1-b]quinazolin-6-one	71.5	C_11_H_11_N_3_O_2_	217	3.9
*Piper retrofractum* Vahl.	0.87	18.59	Caryophyllene	92.1	C_15_H_24_	204	9.4
		20.52	Pentadecane	96.7	C_15_H_32_	212	7.4
		19.42	1,4,7,-Cycloundecatriene, 1,5,9,9-tetramethyl-, Z,Z,Z-	95.0	C_15_H_24_	204	6.8
		24.78	8-Heptadecene	96.0	C_17_H_34_	238	6.8
		25.06	Heptadecane	96.6	C_17_H_36_	240	6.1

## Discussion

Secondary metabolites of plants in particular phenolics and flavonoids are generally involved in defence against oxidative stress-related degenerative disorders. In addition, several reports have shown a correlation between the consumption of plant-derived antioxidants and numerous health benefits in humans [19, 20]. Polyphenolic compounds are the most abundant groups of plant metabolites that structurally possess an aromatic benzene ring with one or more hydroxyl constituents. The antioxidant activity of phenolic compounds is mainly due to their ability to act as reducing agents, hydrogen donors, singlet and triplet oxygen quenchers, and metal chelators [16, 20]. Flavonoids, the major class of polyphenolic compounds, have a common basic structure that consists of two aromatic rings linked with a heterocyclic pyrane ring. The compounds are known to have antioxidant activity by acting as effective scavengers of various free radicals as well as singlet oxygen [20]. The high phenolic and flavonoid contents found in some remedies, such as THP-R010, THP-R014, THP-R015, THP-R016, THP-R017, and THP-R019, may indicate their antioxidant activities and support their acclaim as bracers.

To confirm the beneficial effects on antioxidant abilities, the water extracts of the traditional remedies were primarily assessed for their ability to be either preventive antioxidants, demonstrated by MCA or chain-breaking antioxidants, which were tested using both the single electron transfer mechanism (SET)-based assay (FRAP) and mixed-mode-based assays (DPPH and ABTS scavenging activities) [21, 22]. Metal-mediated formation of hydroxyl radical by the well-known redox cycling process, Fenton reaction, may lead to protein oxidation, DNA damage, and lipid peroxidation [23]. Among the transition metals, ferrous ion is well-recognized as a potential catalysing agent. Hence, ferrous ion chelators may offer protective effects for oxidative damage. This result suggested that the chelating properties of the extracts could be due to the presence of compounds other than phenolics and flavonoids. Previous studies showed that polysaccharides from some medicinal plants [24] possess their abilities to chelate metal ion. It must be noted that the water extract prepared from THP-R014 with relatively high phenolic and flavonoid contents also showed good chelation of ferrous ion. It has been reported that plant-derived compounds, including phenolic acids, flavonoid quercetin, and phenolic glycosides with functional groups of hydroxyl, sulfhydryl, carbonyl, and phosphate, show chelating activity on transition metal ions, including ferrous ion [25].

According to these findings, it should also be noted that phenolic compounds in the herbal remedies are the major contributor to antioxidant capacities. However, this is apparently because of both flavonoid and non-flavonoid phenolics. This result is also similar to the previous studies that found a stronger correlation between the total phenolic content and ABTS assay than the DPPH assay [26, 27]. Both ABTS and DPPH assays were considered mixed-mode methods, and their radicals can be neutralized by SET and HAT mechanisms [21]. ABTS radical cation is applicable to both hydrophilic and lipophilic systems [28], while the radical produced in the DPPH assay is appropriate for hydrophobic systems [29]. Moreover, it has been reported that the difference between antioxidant capacities evaluated by the two methods may be affected by the pigment in the tested extracts [28]. There was a strong association between the antioxidant capacities measured by the ABTS/DPPH assays and FRAP method, which is based on the SET mechanism. This may be explained by the observation that a redox potential of the ferrous/ferric couple is comparable to that of ABTS/ABTS^+•^ redox coupling with values approximately quoted as approximately 0.77 V and 0.68 V, respectively [30]. Therefore, as expected, extracts obtained from polyherbal remedies had similar antioxidant capacities detected by ABTS/DPPH and FRAP assays, which should lead to a significant correlation between the results.

Based on the initial screening, eight effective formulation extracts were selected and tested for their superoxide scavenging activities. Superoxide anion radicals (O_2_^•-^) are mainly generated by activated phagocytes and the mitochondrial electron-transport chain of aerobic respiration, although O_2_^•-^ is not very reactive damaging agents for DNA and polyunsaturated fatty acids in lipids. The physiological radicals play an important role as a precursor of more reactive species, especially hydroxyl radicals that initiate lipid peroxidation and induce several pathophysiological processes [31]. The measurement of the antioxidant capacity of the effective remedy, THP-R016, was further confirmed using the HAT-based assay. The ability of THP-R016 water extract to capture endogenous radicals, such as peroxyl radicals that mediated the oxidation of biological targets, was tested using the ORAC assay. Although, the reaction mechanism of the test is more complex than that of the mixed-mode- and SET-based methods, it has the advantage of acting on physiological radicals [22].

According to the literature, this Thai traditional rejuvenating formulation, THP-R016, is a preparation consisting of 11 medicinal plants, which are *Allium sativum*, *Alpinia galanga*, *Cyperus rotundus*, *Maerua siamensis*, *Piper retrofractum*, *Phyllanthus emblica*, *Terminalia arjuna*, *Terminalia bellirica*, *Terminalia citrina*, *Tinospora crispa*, and *Zingiber officinale*. Except for *M. siamensis*, all medicinal plants have been recorded to possess notable antioxidant capacity. Previous studies demonstrated that piperoside isolated from *P. retrofractum* exhibited moderate peroxyl radical scavenging and reducing potential activity [32], while *Terminalia citrina* was found to display protective effects against oxidant-induced Heinz body formation [33], which contributes to anaemia. Reported active constituents, including total oligomeric flavonoid fractions, nootkatone, aristolone, solavetivone, and orientin obtained from *Cyperus rotundus*, have protective effects against lipid peroxidation, DNA damage, antihemolytic activity, and anticancer treatment for erythroleukaemia cells, which might be correlated with their high antioxidant activities [34]. Earlier experiments have also demonstrated that *Alpinia galangal* exerts in vitro antimelanogenesis activity [35] and in vivo antiamnesiac activity through its antioxidant properties [36]. In addition to their well-described in vitro antioxidant activities, several in vivo studies demonstrated that consumption of *Allium sativum* [37], *Zingiber officinale* [38], *Terminalia arjuna* [39], and *Tinospora crispa* [40] extracts led to significant increases in antioxidant enzymes, such as superoxide dismutase, glutathione peroxidase, and catalase, and suppressed glutathione depletion and lipid peroxidation. It should be noted that the herbal components of THP-R016, *Terminalia bellirica* and *Phyllanthus emblica*, are components of Ayurvedic polyherbal formulas *Charaka Samhita* [8] and *Triphala* [41], which were used as anti-hyperglycemic and rejuvenation agents, respectively.

## Conclusions

From the present study, our findings provide evidence that the water extracts of folkloric polyherbal formulations, especially THP-R016, are a potential source of natural antioxidants, justifying their uses as a health tonic in folk medicines. The extracts exhibited notable free radical scavenging that may be due to the contribution of phenolic and flavonoid contents. However, further investigations need to be performed to measure the antioxidant compounds or determine the in vivo biological activities of these extracts, which are being investigated by our group. This information would be helpful for applying THP-R016 as a traditional-based antioxidant in therapeutic drugs for the implications of human health.

## Additional file

Additional file 1: **Table S1.** Ingredients and proportions of Thai traditional polyherbal formulation used as rejuvenators. (DOCX 55 kb)

## Abbreviations

ABTS: 2,2′-azino-bis (3-ethylbenzothiazoline-6-sulfonic acid)
DPPH: 2,2-diphenyl-1-picrylhydrazyl
MCA: Metal-chelating activity
MTT: Methyl thiazol tetrazolium
TFC: Total flavonoid content
THP: Traditional herbal preparation
TPC: Total phenolic content
TPTZ: Tripyridyl triazine
