# Supplementary material for: Antioxidant capacities and total phenolic contents of 20 polyherbal remedies used as tonics by folk healers in Phatthalung and Songkhla provinces, Thailand
Source: BMC Complement Altern Med. 2018 Feb 21;18:73. doi: 10.1186/s12906-018-2131-y (PMC5822645; doi:10.1186/s12906-018-2131-y)
Supplement: Supplementary file 1 — Table S1. Ingredients and proportions of Thai traditional polyherbal formulation used as rejuvenators. (DOCX 55 kb) [file 12906_2018_2131_MOESM1_ESM.docx]

**Table S1** Ingredients and proportions of Thai traditional polyherbal formulation used as rejuvenators

Remedies Herbal components (Family) Parts used Proportions: Materia medica

(Weight basis) Voucher No.

THP-R001 *Allium sativum* L. (Amaryllidaceae) Bulb 3 MTM08-04

*Cyperus rotundus* L. (Cyperaceae) Rhizomes 1 MTM08-33

*Morinda citrifolia* L. (Rubiaceae) Fruit 13 MTM08-65

*Piper nigrum* L. (Piperaceae) Fruit 1 MTM08-78

*Piper retrofractum* Vahl (Piperaceae) Fruit 1 MTM08-79

*Tinospora crispa* (L.) Hook. f. & Thomson (Menispermaceae) Stem 6 MTM08-95

*Zingiber officinale* Roscoe (Zingiberaceae) Rhizomes 1 MTM08-98

THP-R002 *Boesenbergia rotunda* (L.) Mansf. (Zingiberaceae) Rhizomes 1 MTM08-18

*Curcuma longa* L. (Zingiberaceae) Rhizomes 1 MTM08-30

*Curcuma zedoaria* (Christm.) Roscoe (Zingiberaceae) Rhizomes 1 MTM08-31

*Cyperus rotundus* L. (Cyperaceae) Rhizomes 1 MTM08-33

*Maclura cochinchinensis* (Lour.) Corner (Moraceae) Wood 1 MTM08-56

*Piper nigrum* L. (Piperaceae) Fruit 1 MTM08-78

*Zingiber montanum* (J. Koenig) Link ex A. Dietr.

(Zingiberaceae) Rhizomes 1 MTM08-97

THP-R003 *Amomum testaceum* Ridl. (Zingiberaceae) Fruit 1 MTM08-09

*Angelica dahurica* (Hoffm.) Benth. & Hook. f. ex Franch & Sav. Root 1 MTM08-11

(Apiaceae)

*Ardisia polycephala* Wall. ex A. DC. (Primulaceae) Fruit 1 MTM08-13

*Aristolochia* sp. (Aristolochiaceae) Root 1 MTM08-14

*Atractylodes lancea* (Thunb.) DC. (Asteraceae) Rhizomes 1 MTM08-16

*Clausena excavata* Burm. f. (Rutaceae) Root 1 MTM08-27

*Cuminum cyminum* L. (Apiaceae) Fruit 1 MTM08-29

*Curcuma zedoaria* (Christm.) Roscoe (Zingiberaceae) Rhizomes 1 MTM08-31

*Cyperus rotundus* L. (Cyperaceae) Rhizomes 1 MTM08-33

*Leonurus sibiricus* L. (Lamiaceae) Whole plant 1 MTM08-52

*Lepidium sativum* L. (Brassicaceae) Seed 1 MTM08-53

*Micromelum falcatum* Lour. (Rutaceae) Wood 1 MTM08-62

*Myristica fragrans* Houtt. (Myristicaceae) Aril, Seed 2 MTM08-66

*Nigella sativa* L. (Ranunculaceae) Seed 1 MTM08-68

*Petroselinum crispum* (Mill.) Nyman ex A.W. Hill (Apiaceae) Fruit 1 MTM08-71

*Pimpinella anisum* L. (Apiaceae) Fruit 1 MTM08-74

*Piper cubeba* L. f. (Piperaceae) Fruit 1 MTM08-76

*Pistacia integerrima* Stew. ex Brandis (Pistaciaceae) Gall 1 MTM08-81

**Table S1** (*Continued*)

Remedies Herbal components (Family) Parts used Proportions: Materia medica

(Weight basis) Voucher No.

THP-R003 *Syzygium aromaticum* (L.) Merr. & L. M. Perry (Myrtaceae) Flower 1 MTM08-89

(Continued) *Terminalia chebula* Retz. (Combretaceae) Gall 1 MTM08-92

*Tinospora crispa* (L.) Hook. f. & Thomson (Menispermaceae) Stem 1 MTM08-95

*Zanthoxylum rhetsa* (Roxb.) DC. ( Rutaceae) Fruit 1 MTM08-96

THP-R004 *Aegle marmelos* (L.) Corrêa ex Roxb. (Rutaceae) Fruit 1 MTM08-01

*Amomum testaceum* Ridl (Zingiberaceae) Fruit 1 MTM08-09

*Angelica sinensis* (Oliv.) Diels (Apiaceae) Root 1 MTM08-12

*Carthamus tinctorius* L. (Asteraceae) Flower 1 MTM08-23

*Cinnamomum bejolghota* (Buch.-Ham.) Sweet (Lauraceae) Stem bark 1 MTM08-24

*Clausena excavata* Burm. f. (Rutaceae) Root 1 MTM08-27

*Cyperus rotundus* L. (Cyperaceae) Rhizomes 1 MTM08-33

*Ferula assa-foetida L. (Apiaceae)* Oleo-gum resin 1 MTM08-43

*Glycyrrhiza glabra* L. (Fabaceae) Root 1 MTM08-46

*Myristica fragrans* Houtt. (Myristicaceae) Aril, Fruit 2 MTM08-66

*Piper nigrum* L. (Piperaceae) Fruit 1 MTM08-78

*Syzygium aromaticum* (L.) Merr. & L. M. Perry (Myrtaceae) Flower 1 MTM08-89

THP-R005  *Aegle marmelos* (L.) Corrêa ex Roxb. (Rutaceae) Fruit 1 MTM08-01

*Anethum graveolens* L. (Apiaceae) Fruit 1 MTM08-10

*Angelica dahurica* (Hoffm.) Benth. & Hook. f. ex Franch & Sav.

(Apiaceae) Root 1 MTM08-11

*Angelica sinensis* (Oliv.) Diels (Apiaceae) Root 1 MTM08-12

*Artemisia annua* L.(Asteraceae) Leaf 1 MTM08-15

*Atractylodes lancea* (Thunb.) DC. (Asteraceae) Rhizomes 1 MTM08-16

*Cananga odorata* (Lam.) Hook. f. & Thomson (Annonaceae) Flower 1 MTM08-22

*Cinnamomum bejolghota* (Buch.-Ham.) Sweet (Lauraceae) Bark 1 MTM08-24

*Cinnamomum parthenoxylon* (Jack) Meisn. (Lauraceae) Wood 1 MTM08-25

*Cuminum cyminum* L. (Apiaceae) Fruit 1 MTM08-29

*Curcuma zedoaria* (Christm.) Roscoe (Zingiberaceae) Rhizomes 1 MTM08-31

*Cyperus rotundus* L. (Cyperaceae) Rhizomes 1 MTM08-33

*Dracaena cochinchinensis* (Lour.) S. C. Chen (Asparagaceae) Wood 1 MTM08-38

*Excoecaria agallocha* L. (Euphorbiaceae) Wood 1 MTM08-42

*Foeniculum vulgare* Mill. (Apiaceae) Fruit 1 MTM08-45

**Table S1** (*Continued*)

Remedies Herbal components (Family) Parts used Proportions: Materia medica

(Weight basis) Voucher No.

THP-R005 *Jasminum sambac* (L.) Aiton (Oleaceae) Flower 1 MTM08-49

(Continued) *Kaempferia galanga* L. (Zingiberaceae) Rhizomes 1 MTM08-50

*Lepidium sativum* L. (Brassicaceae) Seed 1 MTM08-53

*Ligusticum chuanxiong* Hort (Apiaceae) Rhizomes 1 MTM08-55

*Magnolia champaca* (L.) Baill. Ex Pierre var. *champaca* Flower 1 MTM08-58

(Magnoliaceae)

*Mammea siamensis* (Miq.) T. Anderson (Calophyllaceae) Flower 1 MTM08-59

*Mesua ferrea* L. (Calophyllaceae) Flower 1 MTM08-61

*Mimusops elengi* L. (Sapotaceae) Flower, Wood 2 MTM08-63

*Nelumbo nucifera* Gaertn. (Nelumbonaceae) Flowers 1 MTM08-67

*Nigella sativa* L. (Ranunculaceae) Seed 1 MTM08-68

*Piper interruptum* Opiz (Piperaceae) Stem 1 MTM08-77

*Piper retrofractum* Vahl (Piperaceae) Fruit 1 MTM08-79

*Piper sarmentosum* Roxb. (Piperaceae) Root 1 MTM08-80

*Plumbago indica* L. (Plumbaginaceae) Root 1 MTM08-82

*Santalum album* L.( Santalaceae) Wood 1 MTM08-86

*Zingiber officinale* Roscoe (Zingiberaceae) Rhizomes 1 MTM08-98

THP-R006 *Alstonia scholaris* (L.) R. Br. (Apocynaceae) Bark 1 MTM08-07

*Cyperus rotundus* L. (Cyperaceae) Rhizomes 1 MTM08-33

*Diospyros rhodocalyx* Kurz (Ebenaceae) Bark 1 MTM08-37

*Prismatomeris tetrandra* (Roxb.) K. Schum. (Rubiaceae) Whole plant 1 MTM08-83

*Senna alata* (L.) Roxb. (Fabaceae) Leaf 1 MTM08-87

*Streblus asper* Lour. (Moraceae) Seed 1 MTM08-88

THP-R007 *Carthamus tinctorius* L. (Asteraceae) Flower 1 MTM08-23

*Morinda citrifolia* L. (Rubiaceae) Leaf 1 MTM08-65

*Ocimum tenuiflorum* L. (Lamiaceae) Whole plant 1 MTM08-69

*Pandanus amaryllifolius* Roxb. (Pandanaceae) Leaf 1 MTM08-70

*Piper sarmentosum* Roxb. (Piperaceae) Root 1 MTM08-80

THP-R008  *Aegle marmelos* (L.) Corrêa ex Roxb. (Rutaceae) Fruit 1 MTM08-01

*Anethum graveolens* L. (Apiaceae) Fruit 1 MTM08-10

*Angelica dahurica* (Hoffm.) Benth. & Hook. f. ex Franch & Sav.
(Apiaceae) Root 1 MTM08-11

*Angelica sinensis* (Oliv.) Diels (Apiaceae) Root 1 MTM08-12

*Artemisia annua* L.(Asteraceae) Leaf 1 MTM08-15

*Atractylodes lancea* (Thunb.) DC. (Asteraceae) Rhizomes 1 MTM08-16

**Table S1** (*Continued*)

Remedies Herbal components (Family) Parts used Proportions: Materia medica

(Weight basis) Voucher No.

THP-R008  *Cinnamomum verum* J. Presl (Lauraceae) Bark 1 MTM08-26

(Continued) *Cuminum cyminum* L. (Apiaceae) Fruit 1 MTM08-29

*Cyperus rotundus* L. (Cyperaceae) Rhizomes 1 MTM08-33

*Foeniculum vulgare* Mill. (Apiaceae) Fruit 1 MTM08-45

*Lepidium sativum* L. (Brassicaceae) Seed 1 MTM08-53

*Ligusticum chuanxiong* Hort (Apiaceae) Rhizomes 1 MTM08-55

*Momordica charantia* L. (Cucurbitaceae) Whole plant 1 MTM08-64

*Nigella sativa* L. (Ranunculaceae) Seed 1 MTM08-68

*Phyllanthus urinaria* L. (Phyllanthaceae) Whole plant 1 MTM08-73

*Piper interruptum* Opiz (Piperaceae) Stem 1 MTM08-77

*Piper retrofractum* Vahl (Piperaceae) Fruit 1 MTM08-79

*Piper sarmentosum* Roxb. (Piperaceae) Root 1 MTM08-80

*Plumbago indica* L. (Plumbaginaceae) Root 1 MTM08-82

*Tinospora crispa* (L.) Hook. f. & Thomson (Menispermaceae) Stem 1 MTM08-95

*Zingiber officinale* Roscoe (Zingiberaceae) Rhizomes 1 MTM08-98

THP-R009  *Azadirachta indica* A. Juss. (Meliaceae) Leaf 1 MTM08-17

*Caesalpinia bonduc* (L.) Roxb. (Fabaceae) Leaf 1 MTM08-20

*Piper nigrum* L. (Piperaceae) Fruit 1 MTM08-78

*Piper retrofractum* Vahl (Piperaceae) Fruit 1 MTM08-79

*Piper sarmentosum* Roxb. (Piperaceae) Fruit 1 MTM08-80

*Plumbago indica* L. (Plumbaginaceae) Root 1 MTM08-82

*Zingiber officinale* Roscoe (Zingiberaceae) Rhizomes 1 MTM08-98

THP-R010  *Aegle marmelos* (L.) Corrêa ex Roxb. (Rutaceae) Fruit 1 MTM08-01

*Coriandrum sativum* L (Apiaceae) Fruit 1 MTM08-28

*Curcuma zedoaria* (Christm.) Roscoe (Zingiberaceae) Rhizomes 1 MTM08-31

*Cyperus involucratus* Rottb. (Cyperaceae) Rhizomes 1 MTM08-32

*Cyperus rotundus* L. (Cyperaceae) Rhizomes 1 MTM08-33

*Gymnopetalum chinense* (Lour.) Merr. (Cucurbitaceae) Fruit 1 MTM08-48

*Mesua ferrea* L. (Calophyllaceae) Flower 1 MTM08-61

*Mimusops elengi* L. (Sapotaceae) Flower 1 MTM08-63

*Nelumbo nucifera* Gaertn. (Nelumbonaceae) Pollen 1 MTM08-67

*Piper interruptum* Opiz (Piperaceae) Stem 1 MTM08-77

*Piper nigrum* L. (Piperaceae) Fruit 1 MTM08-78

*Piper retrofractum* Vahl (Piperaceae) Fruit 1 MTM08-79

*Piper sarmentosum* Roxb. (Piperaceae) Root 1 MTM08-80

**Table S1** (*Continued*)

Remedies Herbal components (Family) Parts used Proportions: Materia medica

(Weight basis) Voucher No.

THP-R010 *Plumbago indica* L. (Plumbaginaceae) Root 1 MTM08-82

(Continued) *Tinospora crispa* (L.) Hook. f. & Thomson (Menispermaceae) Stem 1 MTM08-95

*Zingiber officinale* Roscoe (Zingiberaceae) Rhizomes 1 MTM08-98

THP-R011  *Albizia myriophylla* Benth. (Fabaceae) Stem 1 MTM08-02

*Albizia procera* (Roxb.) Benth. (Fabaceae) Bark 1 MTM08-03

*Cyperus rotundus* L. (Cyperaceae) Rhizomes 1 MTM08-33

*Diospyros rhodocalyx* Kurz (Ebenaceae) Bark 1 MTM08-37

*Elephantopus scaber* L. (Asteraceae) Whole plant 1 MTM08-40

*Glycyrrhiza glabra* L. (Fabaceae) Root 1 MTM08-46

*Kaempferia parviflora* Wall. ex Baker (Zingiberaceae) Rhizomes 1 MTM08-51

*Pueraria candollei* Wall. ex Benth. var. *mirifica* Tuber  1 MTM08-84

(Airy Shaw & Suvat.) Niyomdham (Fabaceae)

*Streblus asper* Lour. (Moraceae) Seed 1 MTM08-88

*Tinospora crispa* (L.) Hook. f. & Thomson (Menispermaceae) Stem 1 MTM08-95

THP-R012 *Alternanthera bettzickiana* (Regel) G. Nicholson Whole plant 1 MTM08-08

(Amaranthaceae)

*Caesalpinia sappan* L. (Fabaceae) Wood 1 MTM08-21

*Maclura cochinchinensis* (Lour.) Corner (Moraceae) Wood 1 MTM08-56

THP-R013  *Aegle marmelos* (L.) Corrêa ex Roxb. (Rutaceae) Fruit 1 MTM08-01

*Boesenbergia rotunda* (L.) Mansf. (Zingiberaceae) Rhizomes 1 MTM08-18

*Caesalpinia sappan* L. (Fabaceae) Wood 1 MTM08-21

*Carthamus tinctorius* L. (Asteraceae) Flower 1 MTM08-23

*Cyperus rotundus* L. (Cyperaceae) Rhizomes 1 MTM08-33

*Morinda citrifolia* L. (Rubiaceae) Fruit 1 MTM08-65

*Piper interruptum* Opiz (Piperaceae) Stem 1 MTM08-77

*Piper retrofractum* Vahl (Piperaceae) Fruit 1 MTM08-79

*Piper sarmentosum* Roxb. (Piperaceae) Root 1 MTM08-80

*Plumbago indica* L. (Plumbaginaceae) Root 1 MTM08-82

*Zingiber officinale* Roscoe (Zingiberaceae) Rhizomes 1 MTM08-98

THP-R014  *Derris scandens* (Roxb.) Benth (Fabaceae) Stem 1 MTM08-34

*Derris trifoliata* Lour. (Fabaceae) Wood 1 MTM08-35

*Lepionurus sylvestris* Blume (Opiliaceae) Wood, Root 2 MTM08-54

*Salacia chinensis* L.(Celastraceae) Wood 1 MTM08-85

THP-R015  *Aegle marmelos* (L.) Corrêa ex Roxb. (Rutaceae) Fruit 1 MTM08-01

**Table S1** (*Continued*)

Remedies Herbal components (Family) Parts used Proportions: Materia medica

(Weight basis) Voucher No.

THP-R015 *Cyperus involucratus* Rottb (Cyperaceae) Rhizomes 1 MTM08-32

(Continued) *Phyllanthus emblica* L. (Phyllanthaceae) Fruit 1 MTM08-72

*Piper interruptum* Opiz (Piperaceae) Stem 1 MTM08-77

*Piper retrofractum* Vahl (Piperaceae) Fruit 1 MTM08-79

*Piper sarmentosum* Roxb. (Piperaceae) Root 1 MTM08-80

*Plumbago indica* L. (Plumbaginaceae) Root 1 MTM08-82

*Terminalia bellirica* (Gaertn.) Roxb. (Combretaceae) Fruit 1 MTM08-91

*Terminalia chebula* Retz. (Combretaceae) Fruit 1 MTM08-92

*Tinospora crispa* (L.) Hook. f. & Thomson (Menispermaceae) Stem 1 MTM08-95

*Zingiber officinale* Roscoe (Zingiberaceae) Rhizomes 1 MTM08-98

THP-R016  *Allium sativum* L. (Amaryllidaceae) Bulb 1 MTM08-04

*Alpinia galanga* (L.) Willd. (Zingiberaceae) Rhizomes 1 MTM08-05

*Cyperus rotundus* L. (Cyperaceae) Rhizomes 1 MTM08-33

*Maerua siamensis* (Kurz) Pax (Capparaceae) Root 1 MTM08-57

*Phyllanthus emblica* L. (Phyllanthaceae) Fruit 1 MTM08-72

*Piper retrofractum* Vahl (Piperaceae) Fruit 1 MTM08-79

*Terminalia arjuna* (Roxb. Ex DC.) Wight & Arn.

(Combretaceae) Fruit 1 MTM08-90

*Terminalia bellerica* (Gaertn.) Roxb. (Combretaceae) Fruit 1 MTM08-91

*Terminalia citrina* (Gaertn.) Roxb. ex Fleming

(Combretaceae) Fruit 1 MTM08-93

*Tinospora crispa* (L.) Hook. f. & Thomson (Menispermaceae) Stem 1 MTM08-95

*Zingiber officinale* Roscoe (Zingiberaceae) Rhizomes 1 MTM08-98

THP-R017 *Albizia procera* (Roxb.) Benth. (Fabaceae) Wood 1 MTM08-03

*Borassus flabellifer* L. (Arecaceae) Root 1 MTM08-19

*Cyperus rotundus* L. (Cyperaceae) Rhizomes 1 MTM08-33

*Diospyros rhodocalyx* Kurz (Ebenaceae) Wood 1 MTM08-36

*Mansonia gagei* J. R. Drumm. ex Prain (Malvaceae) Wood 1 MTM08-60

*Myristica fragrans* Houtt. (Myristicaceae) Seed 1 MTM08-66

*Piper interruptum* Opiz (Piperaceae) Stem 1 MTM08-77

*Piper betle* L. (Piperaceae) Leaf 1 MTM08-75

*Piper nigrum* L. (Piperaceae) Seed, Stem 2 MTM08-78

*Piper retrofractum* Vahl (Piperaceae) Fruit 1 MTM08-79

*Piper sarmentosum* Roxb. (Piperaceae) Root, Leaf 2 MTM08-80

**Table S1** (*Continued*)

Remedies Herbal components (Family) Parts used Proportions: Materia medica

(Weight basis) Voucher No.

THP-R017 *Plumbago indica* L. (Plumbaginaceae) Root 1 MTM08-82

(Continued) *Tinospora crispa* (L.) Hook. f. & Thomson (Menispermaceae) Stem 1 MTM08-95

*Zingiber officinale* Roscoe (Zingiberaceae) Rhizomes 1 MTM08-98

THP-R018 *Albizia myriophylla* Benth. (Fabaceae) Root 1 MTM08-02

*Alstonia macrophylla* Wall. ex G. Don (Apocynaceae) Wood 1 MTM08-06

*Eurycoma longifolia* Jack (Simaroubaceae) Wood/Root 1 MTM08-41

*Prismatomeris tetrandra* (Roxb.) K. Schum. (Rubiaceae) Whole plant 1 MTM08-83

*Tinospora crispa* (L.) Hook. f. & Thomson (Menispermaceae) Stem 1 MTM08-95

THP-R019 *Diospyros toposia* Ham. var. toposioides (King & Gamble) Bark 1 MTM08-37
Phengklai (Ebenaceae)

*Dryopteris  syrmatica* O. kze (polypodiaceae) Rhizomes 1 MTM08-39

*Ficus foveolata* Wall. (Moraceae) Stem 1 MTM08-44

*Goniothalamus macrophyllus* (Blume) Hook. f. & Thomson Root, Wood 2 MTM08-47

(Annonaceae)

THP-R020 *Eurycoma longifolia* Jack (Simaroubaceae) Root, Wood 2 MTM08-41

*Tinospora baenzigeri* Forman (Menispermaceae) Stem 1 MTM08-94

*Tinospora crispa* (L.) Hook. f. & Thomson (Menispermaceae) Stem 1 MTM08-95
